# Cardiac Catheterization and Interventions in Pediatric Patients on ECMO: Analysis of the IMPACT Registry

**DOI:** 10.1016/j.jscai.2025.102570

**Published:** 2025-03-18

**Authors:** Kelsey D. McLean, Gerard R. Martin, Joshua P. Kanter, Kevin F. Kennedy, Shriprasad R. Deshpande

**Affiliations:** a Department of Cardiology, UPMC Children's Hospital of Pittsburgh, University of Pittsburgh Medical Center, Pittsburgh, Pennsylvania; b Department of Pediatric Cardiology, Children's National Hospital, Washington, DC; c Saint Luke's Mid America Heart Institute, Kansas City, Missouri

**Keywords:** catheterization, congenital heart disease, extracorporeal membrane oxygenation, outcomes, risk factors, single ventricle

## Abstract

**Background:**

There are limited published studies on the performance and safety of cardiac catheterization in pediatric patients on extracorporeal membrane oxygenation (ECMO). We sought to understand the utilization and procedural safety of cardiac catheterization in pediatric patients on ECMO. We also aimed to understand differences in the types of interventions and outcomes in patients with single ventricle (SV) physiology compared to those with biventricular physiology.

**Methods:**

Characteristics and outcomes for patients undergoing cardiac catheterization while on ECMO were collected from the American College of Cardiology National Cardiovascular Data Registry: IMPACT (Improving Pediatric and Adult Congenital Treatment) Registry from 2011-2022.

**Results:**

There were 3473 cardiac catheterizations performed on 2980 pediatric patients during the study period. Patients with SV comprised 31.6% of the population. Survival to hospital discharge was 53.2% overall, 45.2% in patients with SV, and 56.8% in patients with 2 ventricles. Interventional procedures occurred in 52.5% of cases. Major adverse events occurred in 11.5%, with arrhythmia, bleeding events, and unplanned cardiac/other surgeries being the commonest. Regression analysis found SV was significantly associated with major adverse events, as were interventional procedures, female sex, non-Hispanic race, diabetes mellitus, procedure status classified as salvage vs elective, and systemic heparinization.

**Conclusions:**

This is the first large, multicenter review of safety and outcomes in pediatric patients undergoing cardiac catheterization while on ECMO. The study identifies common adverse events during the procedure as well as risk factors associated with the same. The presented data can serve for benchmarking, quality assessment as well as potential future research.

## Introduction

The use of extracorporeal membrane oxygenation (ECMO) to provide temporary cardiopulmonary support has continued to increase. It is a therapy that is associated with a high burden of morbidity and mortality.^1^^,^^2^ In pediatric patients, ECMO is commonly utilized in postoperative cardiac surgical patients with low cardiac output syndrome or failure to separate from bypass, in patients with refractory cardiopulmonary resuscitation attempts, and in patients presenting with decompensated heart failure from acute myocarditis.^1^ Outcomes of such ECMO support vary significantly by underlying indications for use. Specifically, congenital heart disease patients supported by ECMO have a higher rate of hospital mortality as seen with registry analysis of the Extracorporeal Life Support Organization.^3^ Such analysis and other studies have identified chromosomal anomalies, single ventricle (SV) physiology, multiple ECMO runs, higher 24-hour ECMO flows, decreased lung compliance, need for plasma exchange, infectious indications, and cardiac indications as risk factors for ECMO mortality.^1^, ^2^, ^3^ Additionally, the presence of residual lesions in those needing ECMO after cardiac surgery has been identified as a potential reason for ECMO nonsurvival. Addressing these residual lesions is important. The use of diagnostic cardiac catheterization in congenital heart disease patients on ECMO can be valuable to assess residual lesions, and hemodynamically significant anatomic issues, and potentially address them. Early catheterization has the potential to reduce time on ECMO as well as potential ECMO survival.^4^, ^5^, ^6^ Although there are single-center and small-scale reports of performance, utility, and safety of cardiac catheterization on pediatric patients supported on ECMO, there are no large multicenter studies addressing this important issue.

The aim of this study was therefore to understand the utilization of cardiac catheterization, interventions performed, and safety of these procedures in pediatric patients supported on ECMO from a multicenter, national database. We also aim to understand differences in the types of interventions and outcomes in patients with SV physiology compared to those with biventricular (BV) physiology.

## Methods

The data for the study were obtained from the National Cardiovascular Data Registry (NCDR) sponsored IMPACT registry (IMproving Pediatric and Adult Congenital Treatments). The details about the registry formation, design, data collection, center participation, and data verification have been described previously.^7^^,^^8^ The centers report patient and procedural data to the registry using standardized data collection forms. The data elements include demographics, diagnostic details, procedural details, findings and complications, and outcomes for the episode of care. The data reporting tools as well as participating centers are available on the NCDR website: https://cvquality.acc.org/NCDR-Home/registries/hospital-registries/impact-registry and https://www.ncdr.com/WebNCDR/impact/home/datacollection. NCDR National Audit Program validates the accuracy of the submitted data on a yearly basis.^8^ To ensure the reliability and validity of an audit, eligible hospitals are given an opportunity to refute the findings, and an interrater reliability assessment of the auditors is built into the program.^8^ All of the definitions used in the study are consistent with the IMPACT registry definitions, including those for cardiac anatomy, primary and nonprimary IMPACT procedures, complications, and outcomes.^8^ The American College of Cardiology has designated Chesapeake Research Review Incorporated as its institutional review board (IRB) of record. Each registry has submitted a protocol to the IRB, which governs all human subject research conducted by that registry. All registry protocols on file have currently been granted a waiver of informed consent.

### Study population

Data from 101 independent sites reporting to the registry were used for this study. Patients aged 0 days to 18 years who were supported with ECMO at the start of their cardiac catheterization were included and data were analyzed. The registry does not delineate the use of veno-venous ECMO vs veno-arterial ECMO and all comers were included.

### Study outcome

The primary outcome of interest was the occurrence of a major adverse event (MAE) whereas the secondary outcome of interest was hospital survival. Occurrence of MAE was defined by having any of the following: death in lab, cardiac arrest, arrhythmia, new heart valve regurgitation, cardiac tamponade, air embolus, embolic stroke, device malposition/thrombosis, device embolization, new dialysis requirement, coronary artery compression, device erosion, postprocedure left ventricular assist device, bleeding, other vascular complication, unplanned surgery, or subsequent catheterization due to complications.

### Statistical analysis

Data are shown as mean ± SD for continuous variables or median (IQR) and n (%) for categorical variables. When comparing SV vs BV patients and patients with MAE and those without MAE we used *t* test or a nonparametric alternative for continuous and χ^2^ test for categorical variables. Using these variables, we ran a multivariable logistic regression model predicting MAE with the following a priori variables: SV defect, age grouping, sex, race/ethnicity, insurance status, heterotaxy, chronic lung disease, diabetes mellitus, preprocedural antiarrhythmic drug, preprocedural antihypertensive drug, procedure status (elective, urgent, emergent, salvage), nonsinus rhythm, interventional procedure performed, prior intubation, systemic heparinization, and inotrope use at the start of the case. Summary statistics are all based on procedure-level data, and for the purposes of statistical analysis, procedures are considered independent observations. However, multivariable models were run with and without clustering procedures within patients in a hierarchical model. Because the clustered and unclustered models produced similar results, we used the clustered model for the final regression model presented. Data are shown as odds ratio and 95% CI. For all comparisons, a *P* < .05 was considered statistically significant for the analysis. All analysis was done with SAS 9.4 (SAS Institute).

## Results

### Patient characteristics and procedures

From January 1, 2011, through June 30, 2022, 3473 diagnostic or interventional cardiac catheterization procedures were performed on 2980 pediatric patients (age 0 days to 18 years) supported by ECMO at 101 centers in the US.

The study population was split into 2 cohorts based on SV physiology (31.6%) and BV physiology (68.4%). The mean age of the SV cohort was 0.77 ± 2.32 years compared with 3.60 ± 5.37 years for the BV cohort. Patients in the SV cohort were more likely to be neonates (47.3% vs 26.6%) or infants (37.6% vs 29.5%). There was a male predominance overall, 59.0% in the SV cohort and 53.5% in the BV cohort. The race distribution was primarily White (49.5%), followed by Black (17.9%), Hispanic (16.4%), other (12.2%), and Asian (4.0%). SV patients were more likely to have a higher proportion of White patients (54.0% vs 47.5%; *P* < .001). The presence of a genetic condition was reported in 14.0% of all patients. Each identified syndrome occurred more frequently in the BV cohort with the exception of heterotaxy, which occurred significantly more frequently in the SV cohort (Supplemental Table S1). Patients in the SV cohort were less likely to have comorbidities than the BV cohort (25.1% vs 32.8%). The payor mix was represented by private and public insurance equally for the entire cohort (40.5% vs 41.0%) with the SV cohort carrying a significantly higher portion of public insurance (44.7%). Patients in the SV cohort were more likely to have had a prior cardiac catheterization or a prior cardiac surgery compared to the BV cohort (27.3% and 35.3% compared with 26.5% and 27.4%). Only 13.6% of the cardiac catheterizations were performed “electively” in total with significant differences in the distribution of the nonelective catheterizations (urgent/emergent/salvage) between the SV and BV cohorts (Table 1).

**Table 1:** Characteristics of pediatric patients undergoing cardiac catheterization while on ECMO from 2011-2022.

Characteristic	Total (N = 3473)	Single ventricle (n = 1096)	Biventricle (n = 2377)	*P* value
Age, y	2.71 ± 4.81	0.77 ± 2.32	3.60 ± 5.37	<.001
Age groups				<.001
Age ≤ 30 d	1151 (33.14%)	518 (47.26%)	633 (26.63%)	
Age 30-365 d	1112 (32.02%)	412 (37.59%)	700 (29.45%)	
Age 1-8 y	729 (20.99%)	136 (12.41%)	593 (24.95%)	
Age 9-17 y	481 (13.85%)	30 (2.74%)	451 (18.97%)	
Sex				.002
Male	1919 (55.25%)	647 (59.03%)	1272 (53.51%)	
Female	1554 (44.75%)	449 (40.97%)	1105 (46.49%)	
Race distribution^a^				<.001
White	1720 (49.52%)	592 (54.01%)	1128 (47.45%)	
Black	622 (17.91%)	190 (17.34%)	432 (18.17%)	
Asian	140 (4.03%)	21 (1.92%)	119 (5.01%)	
Hispanic	568 (16.35%)	171 (15.60%)	397 (16.70%)	
Other	423 (12.18%)	122 (11.13%)	301 (12.66%)	
Insurance payors				.01
Private health insurance	1407 (40.51%)	420 (38.32%)	987 (41.52%)	
Public health insurance	1425 (41.03%)	490 (44.71%)	935 (39.34%)	
Others/missing	641 (18.46%)	186 (16.97%)	455 (19.14%)	
Prior cardiac catheterization	927 (26.77%)	298 (27.26%)	629 (26.54%)	.65
Prior cardiac surgery	1033 (29.91%)	385 (35.26%)	648 (27.43%)	<.001
Procedure status				<.001
Elective	470 (13.56%)	160 (14.63%)	310 (13.06%)	
Urgent	1463 (42.20%)	509 (46.53%)	954 (40.20%)	
Emergency	1262 (36.40%)	353 (32.27%)	909 (38.31%)	
Salvage	272 (7.85%)	72 (6.58%)	200 (8.43%)	
Missing	6	2	4	
Any genetic condition	486 (14.03%)	160 (14.61%)	326 (13.77%)	.51
Any comorbidity	1052 (30.36%)	275 (25.09%)	777 (32.80%)	<.001

Values are mean ± SD or n (%).

ECMO, extracorporeal membrane oxygenation.

a Race designation may be checked for more than 1 race.

Regarding procedural and hemodynamic data acquired while on ECMO, those in the SV cohort had a longer fluoroscopy time compared to the BV cohort (28.1 vs 24.8 minutes; *P* < .001). The rest of the hemodynamics were representative of adequate ECMO support including normal cardiac index (Supplemental Table S2).

### Distribution of catheterization procedures

There was a slight propensity for interventional catheterizations (1822, 52.5%) compared with diagnostic-only catheterizations (1651, 47.5%). Of primary IMPACT procedures (288, 8.3%), proximal pulmonary artery (PA) stenting was performed most frequently (80 in the SV cohort, 97 in the BV cohort), followed by interventions on aortic coarctation (25 in the SV cohort, 19 in the BV cohort). Other primary IMPACT procedures performed in order of decreasing frequency include atrial septal defect closure, aortic valvuloplasty, patent ductus arteriosus (PDA) closure, pulmonary valvuloplasty, and transcatheter pulmonary valve replacement. There were 1534 (44.2%) nonprimary IMPACT procedures performed (Supplemental Table S3), including interventions on the atrial septum/Fontan baffle/atrial baffle which were performed most frequently (34 in the SV cohort, 744 in the BV cohort), followed by stent/device/coil placement on the systemic side (139 in the SV cohort, 125 in the BV cohort), balloon dilation on the venous side (105 in the SV cohort, 104 in the BV cohort), stent/device/coil placement on the venous side (110 in the SV cohort, 96 in the BV cohort), and biopsies (6 in the SV cohort, 195 in the BV cohort). Procedures such as balloon dilation on the systemic side, “other” procedures, and pulmonary vein interventions were performed infrequently (Table 2).

**Table 2:** Procedural information of cardiac catheterizations for patients supported on ECMO.

Procedure	Total (N = 3473)	Single ventricle (n = 1096)	Biventricle (n = 2377)	*P* value
Diagnostic catheterization only	1651 (47.54%)	611 (55.75%)	1040 (43.75%)	<.001
Primary impact procedure	288 (8.29%)	113 (10.31%)	175 (7.36%)	.003
Atrial septal defect closure	21 (0.60%)	0 (0%)	21 (0.88%)	.001
Aortic coarctation procedure	44 (1.27%)	25 (2.28%)	19 (0.80%)	<.001
Aortic valvuloplasty	16 (0.46%)	4 (0.36%)	12 (0.50%)	.57
Pulmonary valvuloplasty	11 (0.32%)	1 (0.09%)	10 (0.42%)	.11
PDA closure	16 (0.46%)	2 (0.18%)	14 (0.59%)	.10
Proximal PA stenting	177 (5.10%)	80 (7.30%)	97 (4.08%)	<.001
Transcatheter pulmonary valve replacement	6 (0.23%)	2 (0.18%)	4 (0.17%)	.90
Other procedure groups	1534 (44.17%)	372 (33.94%)	1162 (48.49%)	<.001
Balloon dilation on the venous side	209 (6.02%)	105 (9.58%)	104 (4.38%)	<.001
Stent/device/coil on the venous side	206 (5.93%)	110 (10.04%)	96 (4.04%)	<.001
Balloon dilation on the systemic side	119 (3.43%)	79 (7.21%)	40 (1.68%)	<.001
Stent/device/coil on the systemic side	264 (7.60%)	139 (12.68%)	125 (5.26%)	<.001
Pulmonary vein interventions	64 (1.84%)	15 (1.37%)	49 (2.06%)	.16
Interventions on atrial septum/Fontan baffle/atrial baffle	778 (22.40%)	34 (3.10%)	744 (31.30%)	<.001
Biopsies	201 (5.79%)	6 (0.55%)	195 (8.20%)	<.001
“Other”	121 (3.48%)	34 (3.10%)	87 (3.66%)	.40

Values are n (%).

ECMO, extracorporeal membrane oxygenation; PA, pulmonary artery; PDA, patent ductus arteriosus.

### Procedural complications and MAE

The most common complications reported included arrhythmia (3.3%), bleeding events (2.7%), unplanned cardiac surgery (1.8%), and other unplanned surgery (1.8%). Complications were similar between the SV and BV cohorts except vascular complications necessitating intervention, which occurred significantly more frequently in the SV cohort compared to the BV cohort (1.6% vs 0.3%; *P* < .001) Overall outcomes demonstrated 6 total deaths in the lab (0.4%), and 41 episodes of cardiac arrest (1.2%). The total cohort had 53.2% survival to hospital discharge, with 45.2% in the SV cohort and 56.8% in the BV cohort (*P* < .001) (Table 3).

**Table 3:** Complications and outcomes reported following cardiac catheterization for pediatric patients supported on ECMO.

Procedural complications	Total (N = 3473)	Single ventricle (n = 1096)	Biventricle (n = 2377)	*P* value
Arrhythmia	116 (3.34%)	42 (3.83%)	74 (3.11%)	.27
New valve regurgitation	7 (0.20%)	3 (0.27%)	4 (0.17%)	.52
Tamponade	15 (0.43%)	2 (0.18%)	13 (0.55%)	.13
Air embolus	4 (0.12%)	3 (0.27%)	1 (0.04%)	.06
Embolic stroke	6 (0.17%)	1 (0.09%)	5 (0.21%)	.43
Device malposition or thrombus	11 (0.32%)	2 (0.18%)	9 (0.38%)	.34
Device embolization (requiring retrieval)	4 (0.12%)	1 (0.09%)	3 (0.13%)	.78
New requirement for dialysis	19 (0.55%)	3 (0.27%)	16 (0.67%)	.14
Coronary artery compression	1 (0.04%)	0 (0.00%)	1 (0.06%)	.50
Erosion	1 (0.04%)	0 (0.00%)	1 (0.06%)	.50
Airway event requiring escalation of care	13 (0.37%)	6 (0.55%)	7 (0.29%)	.26
Event requiring ECMO	28 (0.81%)	14 (1.28%)	14 (0.59%)	.04
Event requiring LVAD	4 (0.12%)	0 (0.00%)	4 (0.17%)	.17
Bleeding event	95 (2.74%)	41 (3.74%)	54 (2.27%)	.01
Vascular complications requiring treatment	25 (0.72%)	18 (1.64%)	7 (0.29%)	<.001
Unplanned cardiac surgery	63 (1.81%)	20 (1.82%)	43 (1.81%)	.97
Unplanned vascular surgery	7 (0.20%)	4 (0.36%)	3 (0.13%)	.14
Unplanned other surgery	62 (1.79%)	27 (2.47%)	35 (1.47%)	.04
Subsequent cardiac catheterization	20 (0.58%)	9 (0.82%)	11 (0.46%)	.19
Outcomes				
Death in lab	6 (0.37%)	2 (0.33%)	4 (0.39%)	.85
Cardiac ​arrest	41 (1.18%)	15 (1.37%)	26 (1.09%)	.49
Alive at discharge	1846 (53.15%)	495 (45.16%)	1351 (56.84%)	<.001

Values are n (%).

ECMO, extracorporeal membrane oxygenation; LVAD, left ventricular assist device.

Next, we assessed the occurrence of MAE. Those with MAE were similar in age, sex, race, and insurance coverage to those who did not experience MAE. MAE occurred more frequently in neonates (155/1151, 13.5%) than other age groups. The history of prior cardiac catheterization or prior cardiac surgery was not significantly different between those with or without MAE. The presence of a genetic syndrome was not predictive of MAE, and no single genetic syndrome was significantly associated with MAE. Overall presence of a comorbidity was not predictive of MAE, but diabetes mellitus and chronic lung disease were more frequently associated with MAE (Supplemental Table S4). A procedure status of salvage procedure was more likely to result in MAE (49/272, 18.0%), compared to less emergent procedure indications (Table 4).

**Table 4:** Characteristics of patients with MAE undergoing cardiac catheterization while on ECMO from 2011 to 2022.

Characteristic	Total (N = 3473)	MAE (n = 400)	No MAE (n = 3073)	*P* value
Age, y	2.71 ± 4.81	2.49 ± 4.71	2.73 ± 4.82	.34
Age groups				.08
Age ≤ 30 d	1151 (33.14%)	155 (38.75%)	996 (32.41%)	
Age 30-365 d	1112 (32.02%)	118 (29.50%)	994 (32.35%)	
Age 1-8 y	729 (20.99%)	74 (18.50%)	655 (21.31%)	
Age 9-17 y	481 (13.85%)	53 (13.25%)	428 (13.93%)	
Weight	14.05 ± 20.51	13.14 ± 20.40	14.17 ± 20.52	.35
Sex				.11
Male	1919 (55.25%)	206 (51.50%)	1713 (55.74%)	
Female	1554 (44.75%)	194 (48.50%)	1360 (44.26%)	
Race distribution^a^				.23
White	1720 (49.52%)	202 (50.50%)	1518 (49.40%)	
Black	622 (17.91%)	78 (19.50%)	544 (17.70%)	
Asian	140 (4.03%)	21 (5.25%)	119 (3.87%)	
Hispanic	568 (16.35%)	52 (13.00%)	516 (16.79%)	
Other	423 (12.18%)	47 (11.75%)	376 (12.24%)	
Insurance payors				.71
Private health insurance	1407 (40.51%)	155 (38.75%)	1252 (40.74%)	
Public health insurance	1425 (41.03%)	171 (42.75%)	1254 (40.81%)	
Others/missing	641 (18.46%)	74 (18.50%)	567 (18.45%)	
Prior cardiac catheterization	927 (26.77%)	99 (24.75%)	828 (26.94%)	.36
Prior cardiac surgery	1033 (29.91%)	119 (29.75%)	914 (29.74%)	.97
Procedure status				<.001
Elective	470 (13.56%)	42 (10.5%)	428 (13.93%)	
Urgent	1463 (42.20%)	153 (38.25%)	1310 (42.63%)	
Emergency	1262 (36.40%)	155 (38.75%)	1107 (36.02%)	
Salvage	272 (7.85%)	49 (12.25%)	223 (7.26%)	
Missing	6	1	5	
Any genetic condition	486 (14.03%)	58 (14.50%)	428 (13.93%)	.78
Any comorbidity	1052 (30.36%)	109 (27.25%)	943 (30.69%)	.15

Values are mean ± SD or n (%).

ECMO, extracorporeal membrane oxygenation; MAE, major adverse event.

a Race designation may be checked for more than 1 race.

We also assessed the occurrence of MAE by procedure. Diagnostic-only procedures on ECMO had a 10.6% (175/1651) rate of MAE. Primary IMPACT procedures had a 14.9% (43/288) rate of MAE, and nonprimary IMPACT interventional procedures had an 11.9% (182/1534) rate of MAE. Of the primary IMPACT interventional procedures performed while on ECMO, only aortic coarctation procedures were found to be significantly more associated with MAE with MAE occurring in 27.3% (12/44) of these procedures. The MAE rates were 25.0% (4/16) for PDA closures, 18.8% (3/16) for aortic valvuloplasty, 14.1% (25/177) for proximal PA stenting, 4.8% (1/21) for atrial septal defect closure, and 0% (0/11) for pulmonary valvuloplasty and (0/6) transcatheter pulmonary valve replacement. Of the nonprimary IMPACT interventional procedures, only stent/device/coil placement on the venous side and “other” interventions were found to be significantly more associated with MAE, with MAE occurring in 16.0% (33/206) and 22.3% (27/121) of these procedures. The MAE rates were 15.1% for balloon dilations on the systemic side, 13.3% for stent/device/coil placement on the systemic side, 12.4% for balloon dilation on the venous side, 11.9% for biopsies, 10.7% for interventions on the atrial septum/Fontan baffle/atrial baffle, and 7.8% for pulmonary vein interventions (Table 5).

**Table 5:** Major adverse events based on procedural category for patients undergoing cardiac catheterization while on ECMO.

Procedure	Total (N = 3473)	MAE (n = 400)	No MAE (n = 3073)	*P* value
Diagnostic-only catheterization	1651 (47.54%)	175 (43.75%)	1476 (48.03%)	.11
Primary impact procedure	288 (8.29%)	43 (10.75%)	245 (7.97%)	.058
Atrial septal defect closure	21 (0.60%)	1 (0.25%)	20 (0.65%)	.33
Aortic coarctation procedure	44 (1.27%)	12 (3.00%)	32 (1.04%)	<.001
Aortic valvuloplasty	16 (0.46%)	3 (0.75%)	13 (0.42%)	.36
Pulmonary valvuloplasty	11 (0.32%)	0 (0%)	11 (0.36%)	.23
PDA closure	16 (0.46%)	4 (1.00%)	12 (0.39%)	.09
PA stenting	177 (5.10%)	25 (6.25%)	152 (4.95%)	.26
Transcatheter pulmonary valve replacement	6 (0.23%)	0 (0%)	6 (0.20%)	.42
Other procedure groups	1534 (44.17%)	182 (45.50%)	1352 (44.0%)	.57
Balloon dilation on the venous side	209 (6.02%)	26 (6.50%)	183 (5.96%)	.67
Stent/device/coil on the venous side	206 (5.93%)	33 (8.25%)	173 (5.63%)	.04
Balloon dilation on the systemic side	119 (3.43%)	18 (4.5%)	101 (3.29%)	.21
Stent/device/coil on the systemic side	264 (7.60%)	35 (8.75%)	229 (7.45%)	.36
Pulmonary vein interventions	64 (1.84%)	5 (1.25%)	59 (1.92%)	.35
Interventions on atrial septum/Fontan baffle/atrial baffle	778 (22.40%)	83 (20.75%)	695 (22.62%)	.40
Biopsies	201 (5.79%)	24 (6.00%)	177 (5.76%)	.85
“Other”	121 (3.48%)	27 (6.75%)	94 (3.06%)	<.001

Values are n (%).

ECMO, extracorporeal membrane oxygenation; MAE, major adverse event; PA, pulmonary artery; PDA, patent ductus arteriosus.

Patients with MAE had high fluoroscopy times, and higher contrast volumes than those without MAE. The rest of the hemodynamics were comparable including cardiac index while supported by ECMO (Supplemental Table S5).

Lastly, we assessed risk factors associated with MAE. Using regression analysis with clustering of the procedures within patients, SV physiology (OR, 1.31; 95% CI, 1.02-1.69) was found to be significantly associated with MAE in patients undergoing cardiac catheterization on ECMO. Other significant risk factors include interventional catheterization, non-Hispanic race, diabetes mellitus, procedure status classified as salvage vs elective, and systemic heparinization (Table 6 and Central Illustration).

**Table 6:** Predictors of major adverse events following cardiac catheterization in pediatric patients on ECMO using a clustered model.

	Odds ratio (95% CI)	*P* value
Single ventricle defect present	1.31 (1.01-1.69)	.04
Age group: ≤30 d vs 9-17 y	1.26 (0.84-1.9)	.26
Age group: 30-365 d vs 9-17 y	0.99 (0.66-1.48)	.95
Age group: 1-8 y vs 9-17 y	0.89 (0.58-1.36)	.58
Sex: male vs female	0.8 (0.64-1.01)	.057
Race/ethnicity: Hispanic vs NH-White	0.67 (0.46-0.96)	.03
Race/ethnicity: NH-Black vs NH-White	1.07 (0.78-1.47)	.67
Race/ethnicity: NH-Asian vs NH-White	1.42 (0.82-2.44)	.21
Race/ethnicity: Other/multiple vs NH-White	1.07 (0.74-1.56)	.71
Insurance: public vs private	1.11 (0.85-1.44)	.46
Insurance: other/none vs private	0.99 (0.71-1.38)	.95
Heterotaxy	1.3 (0.78-2.16)	.31
Chronic lung disease	0.68 (0.42-1.09)	.11
Diabetes	3.88 (1.29-11.65)	.016
Preprocedural antiarrhythmic therapy	1.28 (0.96-1.7)	.09
Preprocedural antihypertensive therapy	0.75 (0.55-1.02)	0.07
Status: urgent vs elective	1.05 (0.69-1.61)	.81
Status: emergency vs elective	1.23 (0.8-1.9)	.34
Status: salvage vs elective	1.73 (1.03-2.93)	.040
Rhythm: nonsinus vs sinus	1.3 (0.95-1.76)	.10
Any procedure vs diagnostic catheterization	1.35 (1.06-1.71)	.015
Previous intubation	1.35 (0.78-2.32)	.28
Systemic heparinization preprocedure	1.46 (1.12-1.9)	.006
Inotrope preprocedure	1.08 (0.8-1.47)	.60

ECMO, extracorporeal membrane oxygenation; NH, non-Hispanic.

**Central Illustration:**
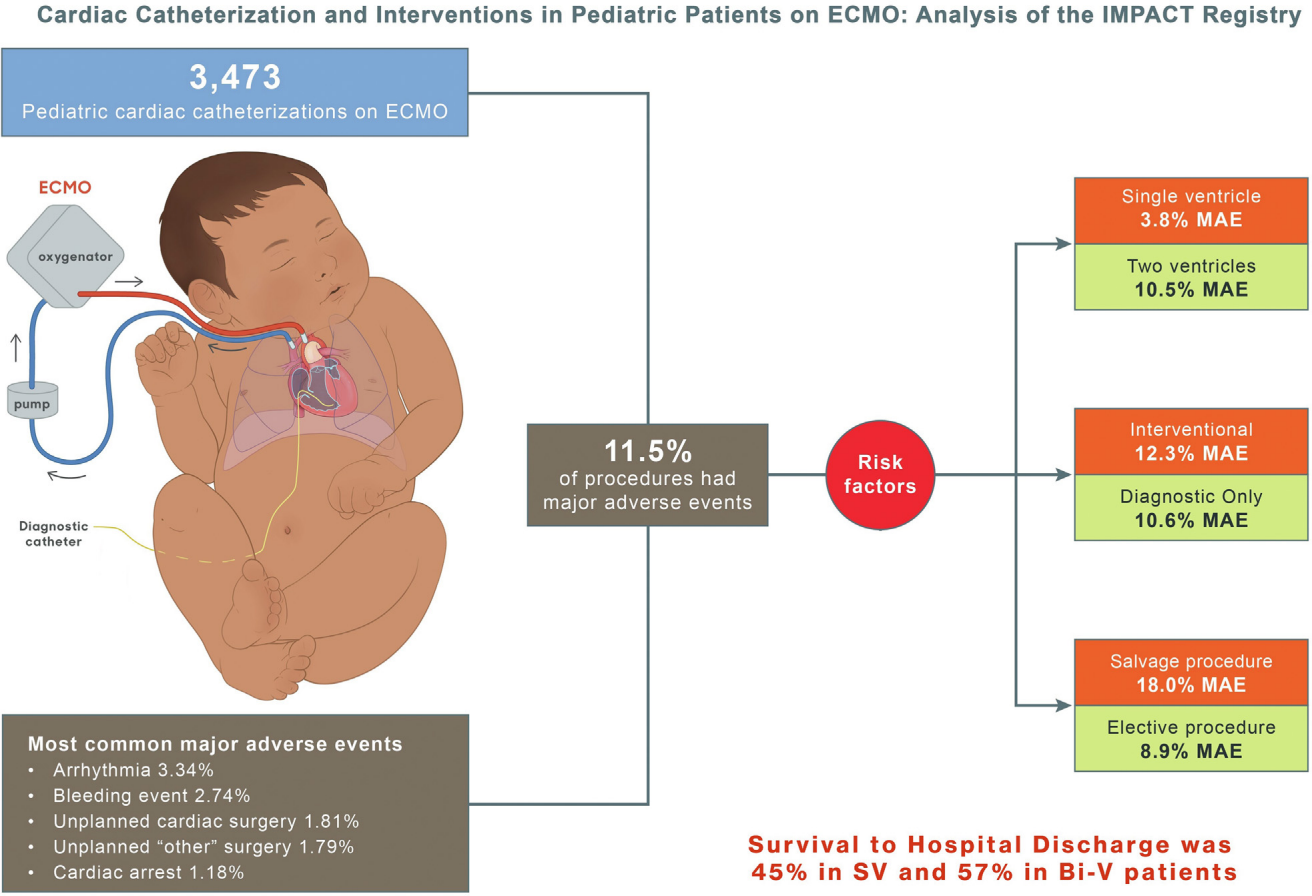
Cardiac catheterizations on pediatric patients on extracorporeal membrane oxygenation (ECMO) had an 11.5% rate of major adverse events (MAE). Risk factors were single ventricle (SV) physiology, interventional procedure, procedure status classified as salvage, non-Hispanic race, diabetes, and systemic heparinization. Bi-V, biventricular; IMPACT registry, IMproving Pediatric and Adult Congenital Treatments.

## Discussion

This is the first large, multicenter study of the performance and procedural safety of cardiac catheterization in pediatric patients supported on ECMO, including those with SV circulation. Overall, this study of over 3400 procedures including over 1000 procedures in SV patients demonstrated a relatively low procedural risk given the clinical acuity of these patients, but about double the risk associated with cardiac catheterization in pediatric patients not on ECMO. The study analyzed diagnostic and procedural catheterizations and identified modifiable and nonmodifiable patient and procedural factors associated with MAE during such catheterizations. Lastly, the study provides benchmarking data for quality improvement, as well as for future studies aimed at further improving the performance of said procedures.

Currently, there are very limited data on the safety of cardiac catheterizations performed in pediatric patients supported on ECMO. Survival to hospital discharge in pediatric patients on ECMO undergoing cardiac catheterization is widely variable and ranges from 29% to 74% with rates of procedural MAE ranging from 0% to 18%.^4^, ^5^, ^6^^,^^9^, ^10^, ^11^, ^12^, ^13^, ^14^ These studies are mostly single-center, small studies with lower survival rates in the earliest studies and improvement in the modern era.^4^, ^5^, ^6^^,^^9^, ^10^, ^11^, ^12^, ^13^, ^14^ In the present study, survival to hospital discharge was 57% in the BV cohort and 45% in the SV cohort, with an overall 11.5% rate of MAE. When compared to the previously described population on ECMO, rates of procedural complications in our study were similar.

Comparing event rates across studies is challenging as there is ambiguity as to what constitutes an MAE. A previous IMPACT report looking at all pediatric patients undergoing catheterization reported an MAE rate of 7%.^15^ The definition of MAE used in our study was very similar to this report. The Congenital Cardiac Catheterization Outcomes Project, which collects data on a similar patient population, has reported an overall adverse event rate of 11%, with hemodynamically significant adverse events occurring in 5% of cases.^16^ Our MAE variables are similar to the hemodynamically significant adverse events reported in that study. Pediatric patients on ECMO are high-risk patients and understandably accrue more risk than the general population, roughly 2 times the amount.

In addition, our study consisted of a significant cohort of SV patient procedures (>1000). This allowed us to assess the impact of an underlying diagnosis of SV on MAE and outcomes in detail. We found that MAE were more likely to occur in SV patients (OR, 1.31; 95% CI, 1.01-1.69; *P* = .04). SV patients who underwent catheterization while on ECMO were younger and more likely to have had a prior cardiac catheterization or cardiac surgery compared to BV patients. This association of SV patients on ECMO and relative risk for MAE is a novel finding.

Major adverse events were also more likely to occur when the procedure involved intervention rather than just diagnostic catheterization. Patients who underwent balloon atrial septostomy while on ECMO, presumably for left atrial hypertension, have been previously described and perhaps carry a unique or different risk stratification.^17^ A 2020 study from the IMPACT registry on atrial septostomy performed in patients on ECMO reported a 54% hospital survival^17^ which approaches our BV cohort survival rate. Not surprisingly, in our group of patients, primary IMPACT interventions including aortic coarctation interventions, PDA closure, aortic valvuloplasty, and proximal PA stenting all had much higher rates of MAE compared to the same interventions performed in patients not on ECMO from the same registry.^15^^,^^18^ However, atrial septal defect closure, pulmonary valvuloplasty, and transcatheter pulmonary valve placement in this population had lower rates of MAE than in prior registry reports. Albeit each of these procedures was performed infrequently in this study group. The majority of interventions performed were nonprimary IMPACT procedures, and individual procedures occurred infrequently which led us to create 8 broad groups of interventions. Overall, interventions performed on ECMO carry a higher risk of MAE than when performed off ECMO.^16^^,^^18^^,^^19^ Based on our grouping of nonprimary IMPACT procedures, only stent/device/coil placement on the venous side and “other” interventions were significantly more associated with MAE, occurring in 16% and 22% of those procedures, respectively. It is unclear why interventions on the right side of the heart or the systemic venous side had higher rates of MAE. With the most common MAE being arrhythmia, perhaps this is more frequent with right-sided procedures that involve intracardiac catheter manipulation. The “other” group is a heterogeneous group of procedures making it difficult to attribute any meaning to this association with MAE. Looking more closely at this group to further risk stratify procedures could be an area of interest for future studies given the high rate of MAE.

Major adverse events also occurred more frequently in patients whose catheterization was categorized as a salvage procedure, with BV patients more frequently undergoing emergent and salvage procedures. This may be a reflection on the acuity of the patient status, lack of preparatory planning, and the urgency of the procedure rather than the performance of the procedure itself. Nonetheless, it underscores the point that any advance planning and avoidance of “last minute” intervention as a rescue, may impact the outcomes of such procedures. That being said, there may be subjectivity in labeling a procedure elective, urgent, or salvage procedure as the data dictionary definitions do not specify a scenario that includes ECMO support. Including further guidance within the data dictionary may help improve consistency in reporting and interpretation of such data.

Systemic heparinization was also significantly associated with MAE. We hesitate to attribute too much clinical significance to this finding given that all patients on ECMO are systemically anticoagulated at baseline. We considered comparing the activated clotting time between the groups but there was a large portion of missing data for that variable. Non-Hispanic race and diabetes mellitus were also more frequent in patients with MAE. Regarding racial disparities, ECMO utilization and outcomes in pediatric patients based on race have been studied previously in large, multicenter studies.^20^^,^^21^ Our cohort had a similar race breakdown as these population-based ECMO utilization studies. A review of the Pediatric Cardiac Critical Care Consortium clinical registry found that Black race and “other” races were associated with increased ECMO utilization during surgical hospitalizations, but racial disparities in outcomes were not explained by differences in ECMO utilization.^20^ Data from the Extracorporeal Life Support Organization Registry demonstrated that Black race and Hispanic ethnicity were independently associated with mortality in children who require cardiac ECMO.^21^ This factor certainly warrants ongoing critical evaluation. We also found the lack of association of MAE with genetic syndromes as noteworthy, although this may be limited by registry reporting accuracy and practice variation of genetic testing and documentation between centers.

Outcomes on ECMO continue to be suboptimal in general, and hospital survival is low. There are data to show that performing catheterizations on ECMO early can improve outcomes.^4^, ^5^, ^6^^,^^14^ Here, we provide data that catheterization on ECMO can be done with relatively low risk in what is considered a high-risk population.

As with all large registry studies, there are inherent limitations such as lack of details and granularity especially around specific cardiac surgeries, inability to account for practice variability among participating and nonparticipating centers, potentially unmeasured confounding variables, and variability in coding and reporting. For example, 0.8% of patients in our study had an event requiring ECMO but also had ECMO in place at the start of the procedure, which we included in our complications but do not necessarily have a good clinical explanation for. The registry has in place rigorous quality assurance standards and periodic data audits to combat some of these limitations. The registry has limited information about the impact of interventions done such as serial hemodynamic data or follow-up surgical interventions. Surgical intervention following catheterization may be to rescue from a complication. However, in other cases, the diagnostic information from the procedure may have guided a surgical intervention allowing for successful recovery. Such detailed information to classify a follow-up surgery after the procedure was not available. Lastly, the timing of the procedure and separation from ECMO could not be ascertained to evaluate the immediate impact of the procedure on the ECMO need. From a statistical standpoint, large registry analysis with multiple comparisons could lead to type I error especially when using a simple *P* value of <.05. We did not adopt overly conservative methods for correction due to the potential for type II error.

## Conclusion

Cardiac catheterization and interventions can be performed safely on pediatric patients supported by ECMO, albeit with higher rates of adverse events compared to patients not on ECMO. We have provided benchmarking data regarding outcomes and identified procedural and patient risk factors for MAE including the presence of SV circulation and the performance of the procedure as a salvage procedure. Further efforts should focus on validation studies, and modification of preprocedural risk factors including avoiding emergent or salvage procedures and optimizing patient outcomes.
