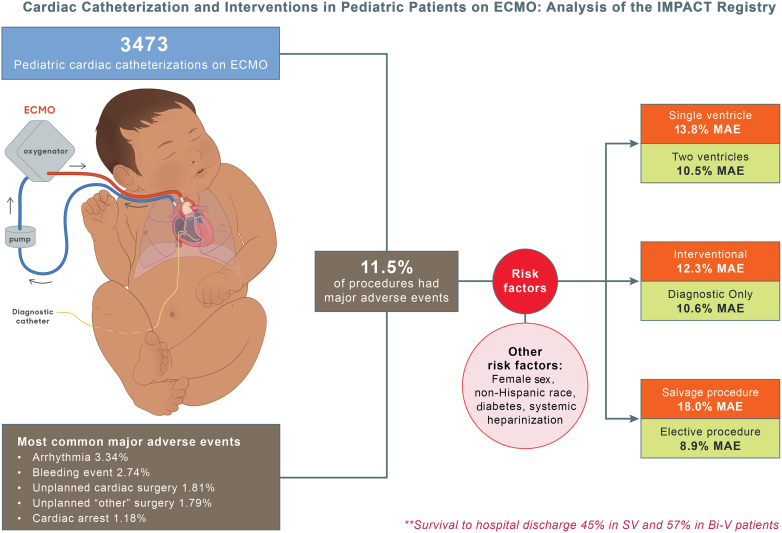# Corrigendum to ‘Cardiac Catheterization and Interventions in Pediatric Patients on ECMO: Analysis of the IMPACT Registry’ [Journal of the Society for Cardiovascular Angiography & Interventions; Volume 4, Issue 3 (2025): 102570]

**DOI:** 10.1016/j.jscai.2026.105308

**Published:** 2026-02-27

**Authors:** Kelsey D. McLean, Gerard R. Martin, Joshua P. Kanter, Kevin F. Kennedy, Shriprasad R. Deshpande

**Affiliations:** a Department of Cardiology, UPMC Children's Hospital of Pittsburgh, University of Pittsburgh Medical Center, Pittsburgh, Pennsylvania; b Department of Pediatric Cardiology, Children's National Hospital, Washington, DC; c Saint Luke's Mid America Heart Institute, Kansas City, Missouri

The authors regret that the Central Illustration figure in this article contains an error.

Current wording: Single ventricle 3.8% MAE

Correct wording: Single ventricle 13.8% MAE

The authors would like to apologise for any inconvenience caused. The corrected figure is included below and contains additional content on other risk factors.

**Figure:**