# Supplementary material for: Cardiac Catheterization and Interventions in Pediatric Patients on ECMO: Analysis of the IMPACT Registry
Source: J Soc Cardiovasc Angiogr Interv. 2025 Mar 18;4(3Part A):102570. doi: 10.1016/j.jscai.2025.102570 (PMC11993852; doi:10.1016/j.jscai.2025.102570)
Supplement: Supplemental Tables [file mmc1.docx]

Supplemental Table S1: Genetic Syndromes and Comorbidities of Pediatric Patients Undergoing Cardiac Catheterization on ECMO

| Characteristic | Total | Single Ventricle | Bi-ventricle | P-Value |
| --- | --- | --- | --- | --- |
|  | n = 3473 | n = 1096 | n = 2377 |  |
| 22q11 Deletion (DiGeorge Syndrome)           Yes           No           Missing | 108 (3.12%) 3355 (96.88%) 10 | 15 (1.37%) 1080 (98.63%) 1 | 93 (3.93%) 2275 (96.07%) 9 | < 0.001 |
| Alagille Syndrome           Yes           No           Missing | 17 (0.49%) 3446 (99.51%) 10 | 1 (0.09%) 1094 (99.91%) 1 | 16 (0.68%) 2352 (99.32%) 9 | 0.022 |
| Congenital Diaphragmatic Hernia           Yes           No           Missing | 33 (0.95%) 3430 (99.05%) 10 | 1 (0.09%) 1094 (99.91%) 1 | 32 (1.35%) 2336 (98.65%) 9 | < 0.001 |
| Down Syndrome           Yes           No           Missing | 99 (2.86%) 3364 (97.14%) 10 | 12 (1.10%) 1083 (98.90%) 1 | 87 (3.67%) 2281 (96.33%) 9 | < 0.001 |
| Heterotaxy           Yes           No           Missing | 161 (4.65%) 3302 (95.35%) 10 | 121 (11.05%) 974 (88.95%) 1 | 40 (1.69%) 2328 (98.31%) 9 | < 0.001 |
| Marfan Syndrome           Yes           No           Missing | 4 (0.12%) 3459 (99.88%) 10 | 0 (0.00%) 1095 (100.00%) 1 | 4 (0.17%) 2364 (99.83%) 9 | 0.173 |
| Noonan Syndrome           Yes           No           Missing | 24 (0.69%) 3439 (99.31%) 10 | 6 (0.55%) 1089 (99.45%) 1 | 18 (0.76%) 2350 (99.24%) 9 | 0.483 |
| Rubella           Yes           No           Missing | 1 (0.03%) 3462 (99.97%) 10 | 1 (0.09%) 1094 (99.91%) 1 | 0 (0.00%) 2368 (100.00%) 9 | 0.141 |
| Trisomy-13                      1                      0           Missing | 3 (0.09%) 3460 (99.91%) 10 | 0 (0.00%) 1095 (100.00%) 1 | 3 (0.13%) 2365 (99.87%) 9 | 0.238 |
| Trisomy-18                      1                      0           Missing | 6 (0.17%) 3457 (99.83%) 10 | 0 (0.00%) 1095 (100.00%) 1 | 6 (0.25%) 2362 (99.75%) 9 | 0.095 |
| Turner Syndrome           Yes           No           Missing | 9 (0.26%) 3454 (99.74%) 10 | 4 (0.37%) 1091 (99.63%) 1 | 5 (0.21%) 2363 (99.79%) 9 | 0.407 |
| Williams-Beuren Syndrome           Yes           No           Missing | 23 (0.66%) 3440 (99.34%) 10 | 0 (0.00%) 1095 (100.00%) 1 | 23 (0.97%) 2345 (99.03%) 9 | 0.001 |
| Cardiomyopathy           Yes           No           Missing | 258 (10.03%) 2315 (89.97%) 900 | 2 (0.25%) 795 (99.75%) 299 | 256 (14.41%) 1520 (85.59%) 601 | < 0.001 |
| Chronic Lung Disease           Yes           No           Missing | 286 (8.26%) 3178 (91.74%) 9 | 73 (6.67%) 1022 (93.33%) 1 | 213 (8.99%) 2156 (91.01%) 8 | 0.020 |
| Coagulation Disorder           Yes           No           Missing | 149 (4.30%) 3315 (95.70%) 9 | 51 (4.66%) 1044 (95.34%) 1 | 98 (4.14%) 2271 (95.86%) 8 | 0.482 |
| Diabetes Mellitus           Yes           No           Missing | 20 (0.58%) 3444 (99.42%) 9 | 3 (0.27%) 1092 (99.73%) 1 | 17 (0.72%) 2352 (99.28%) 8 | 0.109 |
| Hepatic Disease           Yes           No           Missing | 107 (3.09%) 3357 (96.91%) 9 | 25 (2.28%) 1070 (97.72%) 1 | 82 (3.46%) 2287 (96.54%) 8 | 0.062 |
| Renal Insufficiency           Yes           No           Missing | 368 (10.62%) 3096 (89.38%) 9 | 130 (11.87%) 965 (88.13%) 1 | 238 (10.05%) 2131 (89.95%) 8 | 0.104 |
| Seizure Disorder           Yes           No           Missing | 222 (6.41%) 3242 (93.59%) 9 | 94 (8.58%) 1001 (91.42%) 1 | 128 (5.40%) 2241 (94.60%) 8 | < 0.001 |
| Sickle Cell Anemia           Yes           No           Missing | 7 (0.20%) 3456 (99.80%) 10 | 0 (0.00%) 1094 (100.00%) 2 | 7 (0.30%) 2362 (99.70%) 8 | 0.071 |
| Stroke           Yes           No           Missing | 119 (3.43%) 3346 (96.57%) 8 | 44 (4.01%) 1052 (95.99%) | 75 (3.17%) 2294 (96.83%) 8 | 0.202 |

Supplemental Table S2: Procedural information and hemodynamics of pediatric patients undergoing cardiac catheterization while on ECMO

| **Parameter** | **n(%) / Mean (+SD)**  **(n=3473)** | **Single Ventricle**  **(n=1096)** | **Bi-ventricle**  **(n=2377)** | **p-value** |
| --- | --- | --- | --- | --- |
| Access Location |  |  |  |  |
| Venous | 1481 (42.78%) | 404 (37.03%) | 1077 (45.42%) |  |
| Arterial | 657 (18.98%) | 263 (24.11%) | 394 (16.62%) |  |
| Both | 1324 (38.24%) | 424 (38.86) | 900 (37.96%) |  |
| Fluoroscopy time (min) | 25.84 ± 25.41 | 28.10 ± 27.30 | 24.80 ± 24.43 | <0.001 |
| Total contrast volume (ml) | 26.08 ± 31.77 | 27.06 ± 26.65 | 25.61 ± 33.94 | 0.217 |
| Systemic Arterial Saturation (%) | 92.78 ± 10.21 | 88.78 ± 13.22 | 94.72 ± 7.64 | <0.001 |
| Mixed Venous Saturation (%) | 64.41 ± 15.96 | 59.63 ± 16.97 | 66.85 ± 14.84 | <0.001 |
| Systemic Ventricular Systolic Pressure (mmHg) | 77.34 ± 24.00 | 77.42 ± 22.16 | 77.29 ± 25.14 | 0.939 |
| Systemic Ventricular End Diastolic Pressure  (mmHg) | 14.24 ± 7.94 | 12.51 ± 5.43 | 15.34 ± 9.01 | <0.001 |
| Systemic Systolic Blood Pressure (mmHg) | 74.95 ± 18.46 | 73.20 ± 18.20 | 75.91 ± 18.54 | 0.002 |
| Systemic Diastolic Blood Pressure  (mmHg) | 51.23 ± 14.28 | 47.05 ±  12.18 | 53.54 ± 14.83 | <0.001 |
| Systemic Mean Blood Pressure (mmHg) | 59.81 ± 14.41 | 56.51 ± 12.88 | 61.58 ± 14.87 | <0.001 |
| Pulmonary Artery Systolic Pressure (mmHg) | 38.33 ± 21.38 | 33.19 ± 21.08 | 39.88 ±  21.24 | <0.001 |
| Pulmonary Artery Mean Pressure (mmHg) | 24.57 ± 12.80 | 20.89 ± 10.21 | 26.03 ± 13.42 | <0.001 |
| Pulmonary Ventricular Systolic Pressure (mmHg) | 50.79 ± 25.25 | 66.97 ± 23.96 | 48.56 ± 24.61 | <0.001 |
| Pulmonary Vascular Resistance Index [Median (IQR), Wood units*m^2^] | 2.00 (1.10, 3.55) | 1.80 (1.10, 3.20) | 2.10 (1.20, 3.90) | 0.058 |
| Cardiac Index L/min/m^2^ | 4.06 ± 1.93 | 4.09 ± 1.95 | 4.05 ± 1.92 | 0.811 |
| Qp/Qs Ratio | 1.39 ±  1.58 | 1.49 ± 2.23 | 1.35 ± 1.21 | 0.360 |

Supplemental Table S3: Non-Primary IMPACT Procedures Performed on Pediatric Patients on ECMO

| **Group** | **Procedure Type** | **Procedure Name** | **Number performed (%)** |
| --- | --- | --- | --- |
| 1 | Balloon dilation | Conduit: Sano modification (RV to PA valveless conduit) | 10 (0.45) |
| 1 | Balloon dilation | Conduit: Sano modification-with valve (RV to PA valved conduit) | 1 (0.04) |
| 1 | Balloon dilation | Conduit: RA to PA | 1 (0.04) |
| 1 | Balloon dilation | Conduit: RV to PA | 10 (0.45) |
| 1 | Balloon dilation | Intracardiac/septum: Fontan Baffle | 1 (0.04) |
| 1 | Balloon dilation | Intracardiac/septum: Fontan fenestration | 2 (0.09) |
| 1 | Balloon dilation | Pulmonary artery: Central (Proximal left and/or proximal right pulmonary artery including the pulmonary artery bifurcation) | 30 (1.34) |
| 1 | Balloon dilation | Pulmonary artery: Main (Trunk) | 5 (0.22) |
| 1 | Balloon dilation | Pulmonary artery: Peripheral | 19 (0.85) |
| 1 | Balloon dilation | Pulmonary artery: Proximal | 22 (0.98) |
| 1 | Balloon dilation | Systemic vein: Caval vein | 17 (0.76) |
| 1 | Balloon dilation | Systemic vein: Non-Caval vein | 6 (0.27) |
| 1 | Stent re-dilation | Conduit: RV to PA | 1 (0.04) |
| 1 | Stent re-dilation | Pulmonary artery: Central (Proximal left and/or proximal right pulmonary artery including the pulmonary artery bifurcation) | 4 (0.18) |
| 1 | Stent re-dilation | Pulmonary artery: Main (Trunk) | 2 (0.09) |
| 1 | Stent re-dilation | Pulmonary artery: Peripheral | 1 (0.04) |
| 1 | Stent re-dilation | Pulmonary artery: Proximal | 4 (0.18) |
| 1 | Stent re-dilation | Systemic vein: Caval vein | 1 (0.04) |
| 1 | Stent re-dilation | Systemic vein: Non-Caval vein | 2 (0.09) |
| 1 | Balloon dilation | Systemic vein | 3 (0.13) |
| 1 | Balloon dilation | Systemic vein, Caval vein, Superior vena cava | 4 (0.18) |
| 1 | Balloon dilation | Pulmonary artery, Peripheral, Lobar, Left lingula PA | 1 (0.04) |
| 1 | Balloon dilation | Pulmonary artery, Peripheral, Lobar, Left lower PA | 7 (0.31) |
| 1 | Balloon dilation | Pulmonary artery, Peripheral, Lobar, Left upper PA | 2 (0.09) |
| 1 | Balloon dilation | Pulmonary artery, Peripheral, Lobar, Right lower PA | 8 (0.36) |
| 1 | Balloon dilation | Pulmonary artery, Peripheral, Lobar, Right upper PA | 4 (0.18) |
| 1 | Balloon dilation | Pulmonary artery, Peripheral, Sublobar = Segmental, Right | 2 (0.09) |
| 1 | Balloon dilation | Pulmonary artery, Proximal, Left | 28 (1.25) |
| 1 | Balloon dilation | Pulmonary artery, Proximal, Right | 16 (0.72) |
| 1 | Balloon dilation | Systemic vein, Non-Caval vein, Iliac vein | 2 (0.09) |
| 1 | Balloon dilation | Systemic vein, Non-Caval vein, Innominate (Brachiocephalic) | 4 (0.18) |
| 2 | Coil implantation | Pulmonary arteriovenous malformation | 2 (0.09) |
| 2 | Coil implantation | Systemic vein to pulmonary vein collateral | 11 (0.49) |
| 2 | Coil implantation | Systemic vein: Caval vein | 1 (0.04) |
| 2 | Coil implantation | Systemic vein: Non-caval vein | 5 (0.22) |
| 2 | Device implantation | Fontan fenestration | 3 (0.13) |
| 2 | Device implantation | Pulmonary artery | 3 (0.13) |
| 2 | Device implantation | Systemic vein to pulmonary vein collateral | 5 (0.22) |
| 2 | Device implantation | Systemic vein: Caval vein | 2 (0.09) |
| 2 | Device implantation | Systemic vein: Caval vein (Superior vena cava - Right) | 1 (0.04) |
| 2 | Device implantation | Systemic vein: Non-Caval vein | 5 (0.22) |
| 2 | Stent insertion | Conduit: Sano modification (RV to PA valveless conduit) | 21 (0.94) |
| 2 | Stent insertion | Conduit: Sano modification-with valve (RV to PA valved conduit) | 1 (0.04) |
| 2 | Stent insertion | Conduit: RA to PA-pulmonary trunk | 1 (0.04) |
| 2 | Stent insertion | Conduit: RV to PA | 21 (0.94) |
| 2 | Stent insertion | Intracardiac/septum: Fontan Baffle | 1 (0.04) |
| 2 | Stent insertion | Intracardiac/septum: Fontan fenestration | 9 (0.4) |
| 2 | Stent insertion | Pulmonary artery: Main (Trunk) | 26 (1.16) |
| 2 | Stent insertion | Pulmonary artery: Peripheral | 22 (0.98) |
| 2 | Stent insertion | Systemic vein: Caval vein | 19 (0.85) |
| 2 | Stent insertion | Systemic vein: Non-Caval vein | 12 (0.54) |
| 2 | Coil implantation | Systemic vein | 2 (0.09) |
| 2 | Device implantation | Systemic vein | 2 (0.09) |
| 2 | Device implantation | Systemic vein, Caval vein, Inferior vena cava | 1 (0.04) |
| 2 | Device implantation | Systemic vein, Caval vein, Superior vena cava | 1 (0.04) |
| 2 | Stent insertion | Pulmonary artery, Peripheral, Lobar | 1 (0.04) |
| 2 | Stent insertion | Pulmonary artery, Peripheral, Lobar, Right | 1 (0.04) |
| 2 | Stent insertion | Pulmonary artery, Peripheral, Sublobar = Segmental, Left | 2 (0.09) |
| 2 | Stent insertion | Pulmonary artery, Peripheral, Sublobar = Segmental, Right | 3 (0.13) |
| 2 | Stent insertion | Systemic vein, Caval vein, Superior vena cava | 3 (0.13) |
| 3 | Balloon dilation | Conduit: Shunt - systemic-to-pulmonary | 54 (2.42) |
| 3 | Balloon dilation | Systemic artery: Aorta | 6 (0.27) |
| 3 | Balloon dilation | Systemic artery: Systemic artery other than aorta | 15 (0.67) |
| 3 | Stent re-dilation | Conduit: Shunt - systemic-to-pulmonary | 9 (0.40) |
| 3 | Stent re-dilation | PDA | 3 (0.13) |
| 3 | Stent re-dilation | Systemic artery: Aorta | 2 (0.09) |
| 3 | Stent re-dilation | Systemic artery: Systemic artery other than aorta | 1 (0.04) |
| 3 | Balloon dilation | Systemic artery, Systemic artery other than aorta, Coronary artery | 18 (0.81) |
| 3 | Balloon dilation | Systemic artery, Systemic artery other than aorta, Femoral artery | 1 (0.04) |
| 3 | Balloon dilation | Systemic artery, Systemic artery other than aorta, Iliac artery | 1 (0.04) |
| 3 | Balloon dilation | Systemic artery, Systemic artery other than aorta, Innominate artery | 2 (0.09) |
| 4 | Coil implantation | Systemic artery to pulmonary artery collateral | 60 (2.68) |
| 4 | Coil implantation | Systemic artery: Aorta | 2 (0.09) |
| 4 | Coil implantation | Systemic artery: Systemic artery other than aorta | 7 (0.31) |
| 4 | Device implantation | Conduit: Shunt - systemic-to-pulmonary | 1 (0.04) |
| 4 | Device implantation | Coronary artery fistula | 1 (0.04) |
| 4 | Device implantation | Systemic artery to pulmonary artery collateral | 16 (0.72) |
| 4 | Device implantation | Systemic artery: Aorta | 2 (0.09) |
| 4 | Stent insertion | Conduit: LV to aorta | 1 (0.04) |
| 4 | Stent insertion | Conduit: RV to aorta | 1 (0.04) |
| 4 | Stent insertion | Conduit: Shunt - systemic-to-pulmonary | 80 (3.58) |
| 4 | Stent insertion | PDA | 35 (1.57) |
| 4 | Stent insertion | Systemic artery: Aorta | 5 (0.22) |
| 4 | Stent insertion | Systemic artery: Systemic artery other than aorta | 21 (0.94) |
| 4 | Coil implantation | Systemic artery | 2 (0.09) |
| 4 | Device implantation | Systemic artery | 2 (0.09) |
| 4 | Stent insertion | Systemic artery, Aorta, Thoracic aorta | 2 (0.09) |
| 4 | Stent insertion | Systemic artery, Systemic artery other than aorta, Coronary artery | 20 (0.89) |
| 4 | Stent insertion | Systemic artery, Systemic artery other than aorta, Femoral artery | 1 (0.04) |
| 4 | Stent insertion | Systemic artery, Systemic artery other than aorta, Iliac artery | 1 (0.04) |
| 4 | Stent insertion | Systemic artery, Systemic artery other than aorta, Subclavian artery | 1 (0.04) |
| 4 | Stent insertion | Systemic artery, Systemic artery other than aorta, Systemic pulmonary vessel connection | 2 (0.09) |
| 5 | Balloon dilation | Pulmonary vein: Left (Left pulmonary vein [LPV]) | 7 (0.31) |
| 5 | Balloon dilation | Pulmonary vein: Left lower (Left lower pulmonary vein [LLPV]) | 13 (0.58) |
| 5 | Balloon dilation | Pulmonary vein: Left upper (Left upper pulmonary vein [LUPV]) | 11 (0.49) |
| 5 | Balloon dilation | Pulmonary vein: Lingula (Lingular pulmonary vein) | 1 (0.04) |
| 5 | Balloon dilation | Pulmonary vein: Pulmonary venous confluence | 2 (0.09) |
| 5 | Balloon dilation | Pulmonary vein: Right (Right pulmonary vein [RPV]) | 6 (0.27) |
| 5 | Balloon dilation | Pulmonary vein: Right lower (Right lower pulmonary vein [RLPV]) | 11 (0.49) |
| 5 | Balloon dilation | Pulmonary vein: Right middle (Right middle pulmonary vein [RMPV]) | 5 (0.22) |
| 5 | Balloon dilation | Pulmonary vein: Right upper (Right upper pulmonary vein [RUPV]) | 8 (0.36) |
| 5 | Stent insertion | Pulmonary vein: Left (Left pulmonary vein [LPV]) | 2 (0.09) |
| 5 | Stent insertion | Pulmonary vein: Left lower (Left lower pulmonary vein [LLPV]) | 12 (0.54) |
| 5 | Stent insertion | Pulmonary vein: Left upper (Left upper pulmonary vein [LUPV]) | 6 (0.27) |
| 5 | Stent insertion | Pulmonary vein: Pulmonary venous confluence | 9 (0.4) |
| 5 | Stent insertion | Pulmonary vein: Pulmonary venous confluence with left atrium | 1 (0.04) |
| 5 | Stent insertion | Pulmonary vein: Right (Right pulmonary vein [RPV]) | 1 (0.04) |
| 5 | Stent insertion | Pulmonary vein: Right lower (Right lower pulmonary vein [RLPV]) | 4 (0.18) |
| 5 | Stent insertion | Pulmonary vein: Right middle (Right middle pulmonary vein [RMPV]) | 1 (0.04) |
| 5 | Stent insertion | Pulmonary vein: Right upper (Right upper pulmonary vein [RUPV]) | 7 (0.31) |
| 5 | Stent re-dilation | Pulmonary vein: Left lower (Left lower pulmonary vein [LLPV]) | 5 (0.22) |
| 5 | Stent re-dilation | Pulmonary vein: Left upper (Left upper pulmonary vein [LUPV]) | 2 (0.09) |
| 5 | Stent re-dilation | Pulmonary vein: Lingula (Lingular pulmonary vein) | 1 (0.04) |
| 5 | Stent re-dilation | Pulmonary vein: Right middle (Right middle pulmonary vein [RMPV]) | 2 (0.09) |
| 5 | Stent re-dilation | Pulmonary vein: Right upper (Right upper pulmonary vein [RUPV]) | 4 (0.18) |
| 6 | Balloon dilation | Intracardiac/septum: Atrial baffle S/P atrial switch | 1 (0.04) |
| 6 | Balloon dilation | Intracardiac/septum: Atrial septum (Static balloon dilation [without pullback]) | 354 (15.84) |
| 6 | Device implantation | Patent Foramen Ovale (PFO) | 2 (0.09) |
| 6 | Perforation (establishing interchamber and/or intervessel communication) | Atrial septum | 231 (10.34) |
| 6 | Septostomy | Balloon atrial septostomy by pullback (Rashkind) (BAS) | 232 (10.38) |
| 6 | Septostomy | Blade atrial septostomy | 68 (3.04) |
| 6 | Stent insertion | Intracardiac/septum: Atrial baffle S/P atrial switch | 1 (0.04) |
| 6 | Stent insertion | Intracardiac/septum: Atrial septum | 51 (2.28) |
| 6 | Stent re-dilation | Intracardiac/septum: Atrial septum | 2 (0.09) |
| 6 | Septostomy | Septostomy | 71 (3.18) |
| 7 | Biopsy | RV not S/P heart transplant | 112 (5.01) |
| 7 | Biopsy | RV post heart transplant | 84 (3.76) |
| 7 | Biopsy | Site not RV | 4 (0.18) |
| 8 | Balloon valvotomy | Mitral valve | 8 (0.36) |
| 8 | Balloon valvotomy | Tricuspid valve | 4 (0.18) |
| 8 | Device implantation | Perivalvar leak | 2 (0.09) |
| 8 | Device implantation | Ventricular septal defect (VSD) | 5 (0.22) |
| 8 | Other invasive procedures/interventional techniques | Pericardiocentesis - elective | 6 (0.27) |
| 8 | Other invasive procedures/interventional techniques | Pericardiocentesis - emergent | 16 (0.72) |
| 8 | Other invasive procedures/interventional techniques | Pleuracentesis - elective | 16 (0.72) |
| 8 | Other invasive procedures/interventional techniques | Pleuracentesis - emergent | 4 (0.18) |
| 8 | Other invasive procedures/interventional techniques | Snare foreign body | 9 (0.4) |
| 8 | Perforation (establishing interchamber and/or intervessel communication) | Ventricular septum | 1 (0.04) |
| 8 | Stent insertion | Conduit: Other | 6 (0.27) |
| 8 | Stent insertion | Intracardiac/septum: Ventricular septum | 1 (0.04) |
| 8 | Transcatheter implantation of valve | Systemic outflow position | 1 (0.04) |
| 8 | Hybrid Approach "Stage 1" | Application of RPA & LPA bands | 2 (0.09) |
| 8 | Hybrid Approach "Stage 1" | Stent placement in arterial duct (PDA) | 3 (0.13) |
| 8 | Hybrid Approach "Stage 1" | Stent placement in arterial duct (PDA) + application of RPA & LPA bands | 4 (0.18) |
| 8 | Hybrid Approach | Transcardiac balloon dilation | 1 (0.04) |
| 8 | Hybrid Approach | Transcardiac transcatheter device placement | 1 (0.04) |
| 8 | Coil implantation | Coil implantation | 5 (0.22) |
| 8 | Perforation (establishing interchamber and/or intervessel communication) | Perforation (establishing interchamber and/or intervessel communication) | 14 (0.63) |
| 8 | Perforation (establishing interchamber and/or intervessel communication) | Systemic artery, Systemic artery other than aorta, Femoral artery | 1 (0.04) |
| 8 | Transcatheter implantation of valve | pulmonary ventricular inflow position | 1 (0.04) |
| 8 | Transcatheter implantation of valve | Systemic ventricular inflow position | 3 (0.13) |

Groups are described as follows: Group 1-Balloon dilation on venous side, Group 2-Stent/device/coil on venous side, Group 3-Balloon dilation on systemic side, Group 4-Stent/device/coil on systemic side, Group 5-Pulmonary vein interventions, Group 6-Interventions on atrial septum/Fontan baffle/atrial baffle, Group 7-Biopsies, Group 8-“Other”

Supplemental Table S4: Genetic Syndromes and Comorbidities of Pediatric Patients with Major Adverse Events Undergoing Cardiac Catheterization on ECMO

| Characteristic | Total | MAE | No MAE | P-Value |
| --- | --- | --- | --- | --- |
|  | n = 3473 | n = 400 | n = 3073 |  |
| 22q11 Deletion (DiGeorge Syndrome)           Yes           No           Missing | 108  3355  10 | 9 (8.33%) 391 (11.65%) | 99 (91.67%) 2964 (88.35%) 10 | 0.287 |
| Alagille Syndrome           Yes           No           Missing | 17  3446  10 | 3 (17.65%) 397 (11.52%) | 14 (82.35%) 3049 (88.48%) 10 | 0.430 |
| Congenital Diaphragmatic Hernia           Yes           No           Missing | 33  3430  10 | 3 (9.09%) 397 (11.57%) | 30 (90.91%) 3033 (88.43%) 10 | 0.656 |
| Down Syndrome           Yes           No           Missing | 99  3364  10 | 10 (10.10%) 390 (11.59%) | 89 (89.90%) 2974 (88.41%) 10 | 0.647 |
| Heterotaxy           Yes           No           Missing | 161  3302  10 | 25 (15.53%) 375 (11.36%) | 136 (84.47%) 2927 (88.64%) 10 | 0.105 |
| Marfan Syndrome           Yes           No           Missing | 4  3459  10 | 0 (0.00%) 400 (11.56%) | 4 (100.00%) 3059 (88.44%) 10 | 0.469 |
| Noonan Syndrome           Yes           No           Missing | 24  3439  10 | 5 (20.83%) 395 (11.49%) | 19 (79.17%) 3044 (88.51%) 10 | 0.153 |
| Rubella           Yes           No           Missing | 1  3462  10 | 0 (0.00%) 400 (11.55%) | 1 (100.00%) 3062 (88.45%) 10 | 0.717 |
| Trisomy-13                      1                      0           Missing | 3  3460  10 | 0 (0.00%) 400 (11.56%) | 3 (100.00%) 3060 (88.44%) 10 | 0.531 |
| Trisomy-18                      1                      0           Missing | 6  3457  10 | 0 (0.00%) 400 (11.57%) | 6 (100.00%) 3057 (88.43%) 10 | 0.375 |
| Turner Syndrome           Yes           No           Missing | 9  3454  10 | 2 (22.22%) 398 (11.52%) | 7 (77.78%) 3056 (88.48%) 10 | 0.315 |
| Williams-Beuren Syndrome           Yes           No           Missing | 23  3440  10 | 1 (4.35%) 399 (11.60%) | 22 (95.65%) 3041 (88.40%) 10 | 0.278 |
| Any Comorbidity                      1                      0           Missing | 1052  2413  8 | 109 (10.36%) 291 (12.06%) | 943 (89.64%) 2122 (87.94%) 8 | 0.150 |
| Cardiomyopathy           Yes           No           Missing | 258  2315  900 | 21 (8.14%) 228 (9.85%) 151 | 237 (91.86%) 2087 (90.15%) 749 | 0.378 |
| Chronic Lung Disease           Yes           No           Missing | 286  3178  9 | 24 (8.39%) 376 (11.83%) | 262 (91.61%) 2802 (88.17%) 9 | 0.081 |
| Coagulation Disorder           Yes           No           Missing | 149  3315  9 | 15 (10.07%) 385 (11.61%) | 134 (89.93%) 2930 (88.39%) 9 | 0.563 |
| Diabetes Mellitus           Yes           No           Missing | 20  3444  9 | 6 (30.00%) 394 (11.44%) | 14 (70.00%) 3050 (88.56%) 9 | 0.009 |
| Hepatic Disease           Yes           No           Missing | 107  3357  9 | 9 (8.41%) 391 (11.65%) | 98 (91.59%) 2966 (88.35%) 9 | 0.302 |
| Renal Insufficiency           Yes           No           Missing | 368  3096  9 | 46 (12.50%) 354 (11.43%) | 322 (87.50%) 2742 (88.57%) 9 | 0.545 |
| Seizure Disorder           Yes           No           Missing | 222  3242  9 | 22 (9.91%) 378 (11.66%) | 200 (90.09%) 2864 (88.34%) 9 | 0.430 |
| Sickle Cell Anemia           Yes           No           Missing | 7  3456  10 | 1 (14.29%) 398 (11.52%) 1 | 6 (85.71%) 3058 (88.48%) 9 | 0.818 |
| Stroke           Yes           No           Missing | 119  3346  8 | 15 (12.61%) 385 (11.51%) | 104 (87.39%) 2961 (88.49%) 8 | 0.712 |

Supplemental Table S5: Major adverse events based on procedural information of patients undergoing cardiac catheterization while on ECMO

| Parameter | n(%) / Mean (+SD)  (n=3473) | MAE  (n=400) | No MAE  (n=3073) | p-value |
| --- | --- | --- | --- | --- |
| Access Location |  |  |  |  |
| Venous | 1481 (42.78%) | 166 (41.50%) | 1315 (42.79%) |  |
| Arterial | 657 (18.98%) | 86 (21.50%) | 571 (18.58%) |  |
| Both | 1324 (38.24%) | 146 (36.50%) | 1178 (38.33%) |  |
| Fluoroscopy time (minutes) | 25.84 ± 25.41 | 29.67 ± 28.64 | 25.33 ± 24.91 | 0.001 |
| Total contrast volume (ml) | 26.08 ± 31.77 | 30.10 ± 42.75 | 25.55 ± 29.99 | 0.007 |
| Systemic Arterial Saturation(%) | 92.78 ± 10.21 | 92.48 ± 11.56 | 92.81 ± 10.04 | 0.676 |
| Mixed Venous Saturation (%) | 64.41 ± 15.96 | 63.27 ± 17.32 | 64.52 ± 15.82 | 0.477 |
| Systemic Ventricular Systolic Pressure (mmHg) | 77.34 ± 24.00 | 76.45 ± 23.71 | 77.44 ± 24.05 | 0.692 |
| Systemic Ventricular End Diastolic Pressure  (mmHg) | 14.24 ± 7.94 | 14.37 ± 6.82 | 14.23 ± 8.05 | 0.868 |
| Systemic Systolic Blood Pressure (mmHg) | 74.95 ± 18.46 | 74.20 ± 18.30 | 75.04 ± 18.48 | 0.545 |
| Systemic Diastolic Blood Pressure  (mmHg) | 51.23 ± 14.28 | 51.18 ±  14.24 | 51.23 ± 14.29 | 0.961 |
| Systemic Mean Blood Pressure (mmHg) | 59.81 ± 14.41 | 58.94 ± 13.69 | 59.91 ± 14.49 | 0.375 |
| Pulmonary Artery Systolic Pressure (mmHg) | 38.33 ± 21.38 | 40.04 ± 23.17 | 38.17 ±  21.20 | 0.420 |
| Pulmonary Artery Mean Pressure (mmHg) | 24.57 ± 12.80 | 25.12 ± 13.13 | 24.51 ± 12.77 | 0.637 |
| Pulmonary Ventricular Systolic Pressure (mmHg) | 50.79 ± 25.25 | 51.84 ± 25.50 | 50.69 ± 25.24 | 0.679 |
| Pulmonary Vascular Resistance Index [Median (IQR), Wood units*m^2^] | 2.00 (1.10, 3.55) | 1.50 (1.20, 3.40) | 2.00 (1.10, 3.60) | 0.635 |
| Cardiac Index L/min/m^2^ | 4.06 ± 1.93 | 3.88 ± 1.54 | 4.08 ± 1.95 | 0.499 |
| Qp/Qs Ratio | 1.39 ±  1.58 | 1.43 ± 0.88 | 1.39 ± 1.63 | 0.875 |
